# The Etiology of "Return" Cases of Scarlet Fever

**Published:** 1898-10-29

**Authors:** 


### EDITOR'S LETTER-BOX.

[Our Correspondents are reminded that prolixity is a great bar to publication, and that brevity of style and conciseness of statement greatly facilitate early insertion.]

### THE ETIOLOGY OF "RETURN" CASES OF SCARLET FEVER.

Dr. R. Alex. Shannon writes: In your issue of September 24th you refer to Dr. Clement Dukes' comments on Dr. Killick Millard's exhaustive paper upon the etiology of return cases of scarlet fever. You state that Dr. Dukes' theory is that even after a patient has ceased to be himself infectious he may still, if placed in the same room with a recent case, become a carrier of infection, his nasal cavities and fauces becoming saturated with the fresh virus of scarlet fever. In connection with this point, I would desire to know why Dr. Dukes selects the nasal cavities and fauces, and leaves out the lungs with the residual air which they contain, and which may not be expired for some time after leaving the hospital? I have no doubt about the correctness of this theory, but, however this may be, I desire, on the present occasion, to take up the position of defending the patient discharged from the hospital. I quite agree with Dr. Millard that the very greatest attention should be paid to the patient about to be discharged from the hospital, but why is it that when a return case occurs all the suspicion is directed to the discharged patient, when, until the second case occurs, and, indeed, even afterwards, it is, in most cases, admitted by all concerned, medical and lay, that he is absolutely perfect. In fact, my experience is that the more perfect the patient the more likely is there to be a return case from him. I have been medical superintendent of an infectious hospital for more than ten years, and I cannot help thinking that it is not to the patient discharged from the hospital that suspicion should be directed, and I would ask: (1) Is it logical to set down a return case as undoubtedly due to an otorrhœa or rhinorrhœa which a patient suffered from after leaving the hospital, when it frequently happens that a return case occurs without the discharged patient having any otorrhœa or rhinorrhœa? (2) Is it logical to set down a return case as undoubtedly due to a patient not having been detained long enough in hospital, when it frequently happens that a return case occurs after a patient has not only been in hospital but in a convalescent home? (3) Is it logical to set down a return case as undoubtedly due to a patient recently discharged from an infectious hospital, when it frequently happens that a return case occurs (excuse the Irishism) without a patient coming from the hospital at all? Two typical cases of such an occurrence are under my observation at present. (4) Is it the custom when a return case occurs to examine the other members of the family besides the patient recently arrived from the hospital, and, if so, have they always been found to be free from desquamation, &c.? A very able medical officer of health (and I may remark in passing that return cases occur just as frequently in places where the one medical man holds both the office of medical officer of health and that of medical superintendent of the infectious hospital), who has of late had particular opportunity of studying these cases, states in his annual report that, "taking into consideration the number of cases in which scarlet fever appeared again in households after disinfection (?), I am not at all satisfied that our present methods of disinfection of dwellings by sulphur is good, and this doubt is strengthened by some experiments on this subject which have lately been carried out." Evidently this gentleman's suspicions are being removed from the patient and are turning in the direction of the patient's home; and here I would instance the following case which recently occurred: A family were just about to remove to another house when one member fell ill with scarlet fever and was sent into hospital. Soon afterwards the family completed their removal and the house was disinfected. In the course of a week or so fresh occupants came into the vacated house, and one of them failed with scarlet fever and was received into hospital. Would not this case appear to point in an unmistakable manner to infection still lying about the house? There are just one or two more questions which I should like to ask in connection with this subject, and I should be very much obliged if they could be answered:—(1) Has the use of carbolic soap and carbolic oil not been observed to cause a sort of desquamation of the body, which has been mistaken for secondary scarlatinal desquamation? Surgeons who practise antiseptic surgery say that carbolic acid causes desquamation. (2) Is it the custom in our metropolitan hospitals to put "Izal" or any other so-called "disinfectant" into the water for bathing or to oil the patients in any way? (3) Can there be such a thing as a non-poisonous disinfectant?

